# Clear Aligners Treatment of Class III Subdivision with an Extraction of a Lower Bicuspid

**DOI:** 10.3390/ijerph20043550

**Published:** 2023-02-17

**Authors:** Vincenzo D’Antò, Rosa Valletta, Vittoria De Simone, Massimo Pisano, Stefano Martina

**Affiliations:** 1 Department of Neurosciences, Reproductive Sciences and Oral Sciences, University of Naples Federico II, Via Pansini 5, 80131 Naples, Italy; 2 Department of Medicine, Surgery and Dentistry “Scuola Medica Salernitana”, University of Salerno, Via Allende, 84081 Baronissi, Italy

**Keywords:** orthodontics, Class III malocclusion, camouflage, extractions, clear aligners

## Abstract

The aim of this study was to show a case of a Class III subdivision adult patient treated with clear aligners (CA) and the extraction of a lower bicuspid. A 19-year-old male with a class III canine and molar relationship on the right side and a deviation of lower dental midline to the left asked for an aesthetic treatment. He refused orthognathic surgical procedures, so he was offered a camouflage orthodontic treatment with the extraction of lower right first premolar to achieve a canine Class I relationship and to center the lower midline. The treatment was performed with clear aligners and the use of Class III elastics to maintain distal anchorage on the right side during the canine distalization. At the end of the treatment, the occlusal objectives were achieved.

## 1. Introduction

The management of Class III malocclusion has always been a challenge for clinicians [1]. Depending on the age of the patient and the stage of growth, different approaches have been described in the scientific literature. If the patient is of a pre-pubertal age and in early mixed dentition, the aim of Class III treatment is to try re-establishing the normal skeletal growth pattern and improving the occlusal relationship [2]. The most utilized approaches in growing patients are represented by orthopedic therapy with a rapid maxillary expansion and facemask or with other functional appliances [3,4]. When the patient passes the growth peak, dental compensation without correction of the skeletal malocclusion becomes the only possible strategy, or alternatively, if the skeletal discrepancy is too great, it is necessary to wait until the end of growth and seek orthognathic surgery [5]. When the Class III malocclusion is characterized by dental and/or skeletal asymmetries, the treatment can be even more complex [6]. In non-growing subjects with Class III subdivision malocclusion and a midline deviation, combined surgical–orthodontic therapy can be a treatment option, especially if the asymmetry is severe [7,8]. An alternative approach for patients who reject surgery is represented by a compensatory orthodontic treatment associated with tooth extractions [9,10]. The most commonly extracted teeth in treatment with camouflage purposes are bicuspids, but molar and incisor extractions are also described in the literature [11,12].

In recent years, clear aligners have become very popular for orthodontic treatment in adult patients, considering that they are more aesthetic, more comfortable and easier to manage in terms of both emergencies and oral hygiene as compared to braces [13,14]. Today, clear aligners are used to treat different malocclusions [15,16], thanks also to the improvement of auxiliaries and attachments and aligners materials [17,18], which allow the achievement of complex movements, such as rotation or torque [19,20]. However, there is a lack of literature describing the management of extractive cases using clear aligners, particularly with a single extraction in the lower arch. 

The present case report describes the results obtained in an adult patient with a Class III subdivision malocclusion and dental midline deviation, treated with clear aligners and the extraction of a lower bicuspid.

## 2. Case Report

**Figure 1:**
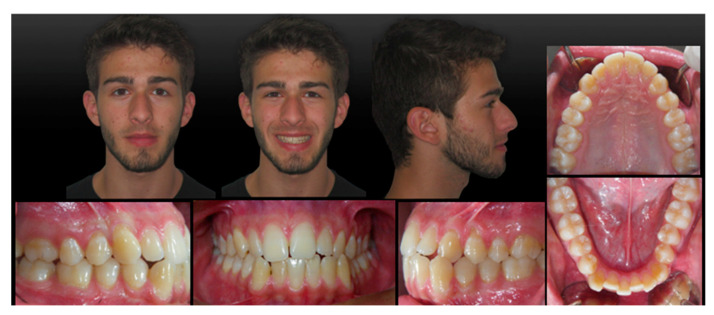
Pretreatment extra- and intra-oral records.

**Figure 2:**
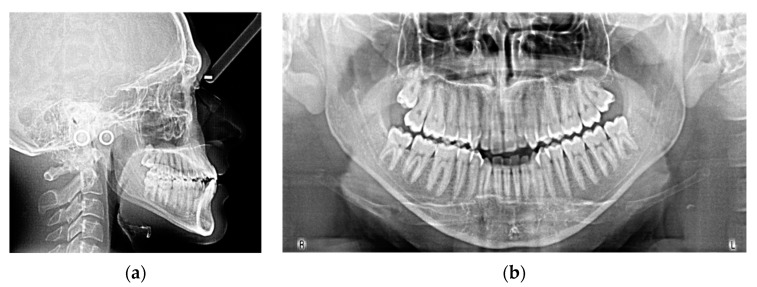
Lateral cephalogram (**a**) and panoramic radiograph (**b**) at the beginning of the treatment.

A 19-year-old man presented for observation with the chief complaint of crowded lower teeth. Extraoral examination showed a Class III skeletal pattern with a nasolabial angle increased and flat cheekbone contouring, showing a slight maxillary hypoplasia; increased lower facial height; and a slight deviation of the chin to the left (Figure 1). Intraoral examination and the analysis of dental casts showed a Class III subdivision malocclusion with molar and canine Class III relationship on the right side and Class I relationship on the left. Moderate mandibular crowding and the crossbite of the canines (1.3–4.3, 2.3–3.3) were noted. The overjet and the overbite were reduced. Bolton analysis showed a discrepancy with an increased anterior Bolton index. The upper dental midline was coincident to the facial midline and the lower dental midline was deviated by 4 mm to the left side (Figure 1). The functional analysis did not detect a CR/CO shift. The panoramic radiograph showed that all permanent teeth were present, including the third molars (Figure 2b). The cephalometric analysis confirmed that the patient had a hyperdivergent growth pattern (SN-GoGn = 37.7°, ANS/PNS-GoGn = 33.3°). The maxillary incisors were slightly proclined, while the mandibular incisors were lingually inclined, but well positioned in relation to the Pogonion (Table 1). The patient was diagnosed with a Class III subdivision right malocclusion and deviated lower dental midline.

The primary treatment objectives were to establish a canine Class I relationship on the right side, to center the lower dental midline with the upper one, to correct the mandibular crowding, and to achieve a normal overjet and overbite.

As a first option, a surgical treatment plan was proposed to the patient to solve mandibular asymmetry and to improve maxillary retrusion and simultaneously achieve occlusal objectives while normalizing the position of the incisors: a maxillary Le Fort I advancement combined with a mandibular bilateral sagittal split osteotomy (BSSO) setback, which involved the extraction of the two mandibular third molars. The patient refused the surgical procedures, so he was presented with orthodontic camouflage treatment. The patient accepted this option and asked for an aesthetic treatment. Hence, the first treatment plan included the use of clear aligners and the extraction of a lower bicuspid (4.4), with the use of Class III elastics on the right side only during the night. Another treatment option was the unilateral distalization of the fourth quadrant teeth by using skeletal anchorage and with the extraction of the lower right third molar (4.8). However, this procedure was longer and less predictable, so the patient chose the treatment with the extraction of the premolar.

**Figure 3:**
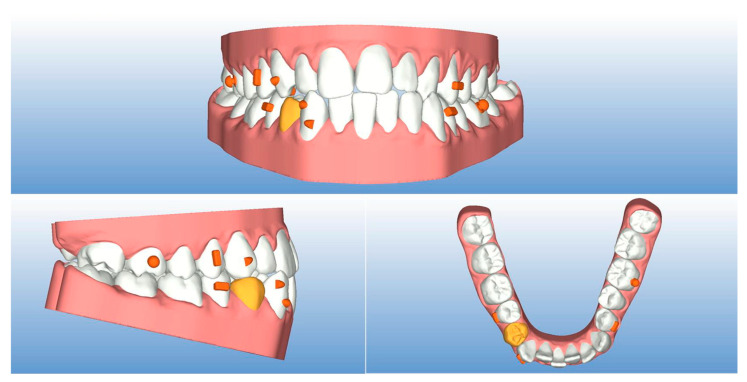
Step 0 of the virtual treatment plan (extracted tooth in yellow, attachments and buttons for elastics in orange).

The lower right bicuspid (4.4) was extracted one week before the patient received the first two aligners, with the aim of taking advantage of the regional acceleratory phenomenon (RAP) [21]. A couple of optimized buccal attachments was used on the lower right cuspid (4.3) to generate a reliable bodily movement toward the extraction space. Attachments were not used on lower incisors to prevent the risk of debonding as the patient was living abroad and could not undergo monthly controls (Figure 3). The patient was instructed to wear a Class III elastic on the right side only for the night. The elastic was attached from a precision cut in the lower right canine area to a bonded button on the upper right first molar, increasing the distal anchorage in the lower right hemiarch. The present case required 20 upper and 44 lower aligners, and changing each pair every ten days was recommended.

**Figure 4:**
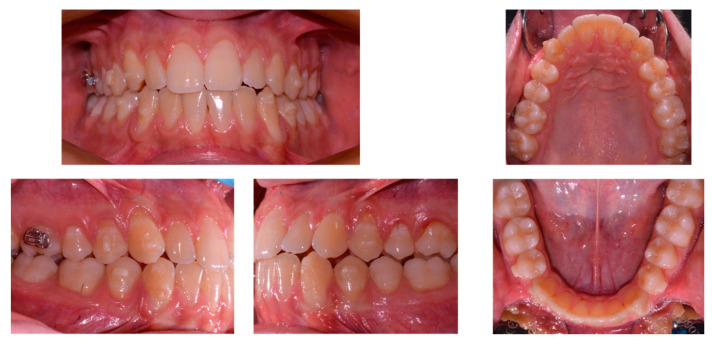
Intraoral records before refinement phase.

At the end of the first treatment phase (after 15 months), an open bite tendency and reduced overjet (due to the increased Bolton index) were present (Figure 4). The finishing phase comprised 15 upper and 10 lower aligners; interproximal reduction between lower front teeth was planned to normalize the Bolton index and further increase the overjet. Palatal extrusion attachments were used on the upper incisors to increase the overbite while maintaining the correct inclination of the incisors. The total treatment time was 20 months. Subsequently, vacuum-formed retainers were delivered to the patient as retention, recommending that they be worn 24 hours a day for the first two months after the end of the active treatment, and then progressively reducing the number of hours until they were to be worn only at night.

**Figure 5:**
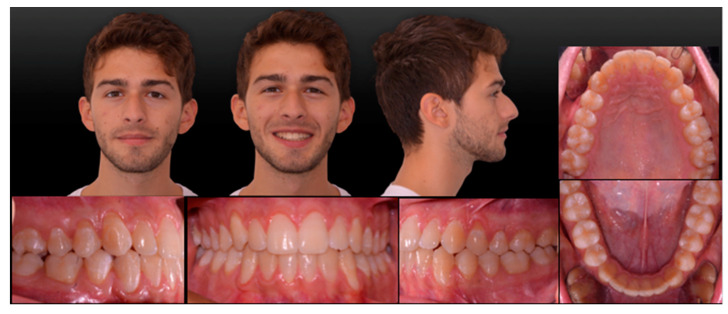
Post-treatment extra- and intra-oral records.

**Figure 6:**
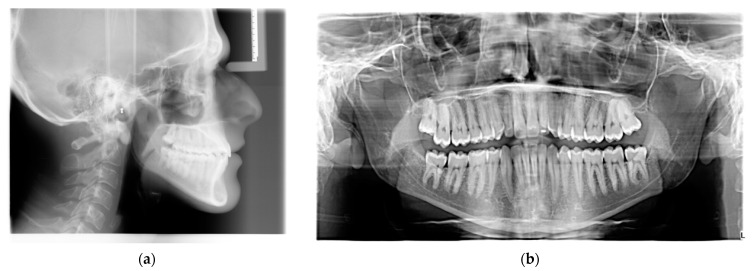
(**a**) Lateral cephalogram and (**b**) panoramic radiograph at the end of the treatment.

**Figure 7:**
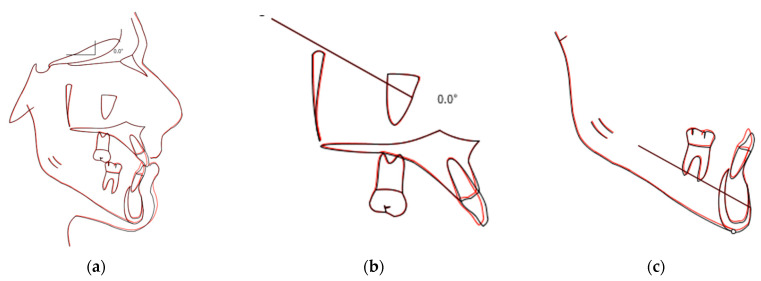
Total (**a**), maxillary (**b**), and mandible (**c**) cephalometric tracing superimposition before (black) and after (red) treatment.

The post-treatment records demonstrated that all the treatment objectives were achieved. Extraoral photographs showed the improvement in facial aesthetics both in the frontal and lateral view, although, obviously, the skeletal deviation of the chin remains unchanged (Figure 4). Intraorally, the dental midlines were coincident with the facial midline, and a canine Class I relationship on both sides was present. The crossbite was successfully corrected and a normal overjet and overbite were achieved (Figure 5). A panoramic radiograph confirmed bodily tooth movement, with the roots of the lower right teeth parallel after treatment (Figure 6b). The final cephalometric radiograph, tracings and superimposition revealed that the inclination of the maxillary incisors did not change, while the mandibular incisors became slightly retroclined (Figure 6). As expected for a non-growing patient, there were no differences in the position of the maxilla or the mandible (Figure 7, Table 1). At the end of the therapy, the patient was satisfied with his dental and facial appearance.

## 3. Discussion

The purpose of this study is to show a Class III subdivision case with lower midline deviation treated with a premolar extraction and clear aligners. Class III asymmetrical malocclusion has always been a biomechanical challenge for orthodontists. When large dentoskeletal discrepancies are present, a combination of orthodontics and orthognathic surgery remains the treatment of choice [22]. If the patient refuses a surgical approach and dentoalveolar compensation or camouflage are possible, therapeutic alternatives are represented by fixed orthodontic treatment with or without extraction, depending on the severity of the malocclusion [23]. Different approaches to Class III subdivision malocclusion have been described in the literature [24,25,26]. Janson et al. [24] reported a case of Class III subdivision malocclusion with lower midline deviation, treated with the extraction of two lower and one upper bicuspids (on the Class I side). Initially, the treatment plan involved four premolar extractions to obtain molar and canine Class I on both sides, including the daily use of bilateral Class III elastics. However, the authors considered that the success of this therapy depended largely on patient compliance, and it increased the risk of treatment failure. Consequentially, they opted for an alternative approach with the extraction of two lower and one upper premolar in order to center the lower dental midline with the upper one and to obtain a molar and canine Class I on the Class I side and a molar Class III and canine Class I relationship on Class III side, with a minimal use of inter-maxillary elastics. Brunetto et al. [25] presented a case of Class III subdivision malocclusion, managed as a non-extractive treatment, with the use of Class III elastics 24 hours per day applied on one side only; however, the long-term use of unilateral inter-maxillary elastics may lead to undesired effects on both arches, such as the extrusion of the teeth involved, resulting in a canting of the occlusal plane [27]. Zimmer et al. [26] demonstrated that treating Class III malocclusion by the isolated extraction of lower premolars could be a valid alternative. In this clinical case, the sagittal and transverse skeletal discrepancy was such that orthodontic camouflage treatment could also be planned, but the amount of deviation of the lower midline made extractive treatment the only possible option. Among the various extraction patterns, a single extraction of a first premolar was chosen to reduce the treatment time and the required inter-arch anchorage. Another possible option would have been the extraction of 4.8 and the distalization of the whole hemiarch to achieve a Class I molar and canine relationship, but the amount of distalization would have been excessive, the distal anchorage needed would have been higher, and the time of the treatment would have been much longer.

In addition, the patient had asked for an aesthetic treatment and had warned that he would have to spend a few months working abroad. The literature showed that clear aligners are more aesthetic, more comfortable, allow better oral hygiene and have fewer emergency visits compared to fixed appliances [13,28,29]. Furthermore, in recent years, researchers have described more and more complex malocclusions treated with aligners with effectiveness and good predictability [30,31]. In light of the above, the choice of aligners was almost mandatory for this patient. On this basis, the treatment option with the extraction of 4.8 and molar distalization was rejected. Indeed, a recent article by Rota et al. [32] demonstrated that the sequential distalization with clear aligners for lower molars mainly determines a tipping movement rather than a true distal translation. Thus, considering that in this case the molar class correction needed a distalization of approximately 5 mm, we assumed that it would be not predictable and, therefore, the only viable therapeutic alternative with clear aligners was the extraction of a lower premolar.

In this case, clear aligners also provided advantages in terms of orthodontic biomechanics. Indeed, the vertical control of the posterior teeth due to the posterior bite-block effect from the aligners’ constant occlusal coverage [33] was able to prevent the undesired effects of the asymmetric use of the Class III elastics, which was even more important in this case, considering the patient’s dolichofacial pattern. Moreover, previous studies showed that the root movements of lower incisors with aligners are more predictable than other teeth because of their reduced root surface area [19,20,34]. The least predictable movement was canine bodily distalization for which an optimized couple of buccal attachments was provided to generate the moment of the couple in order to counterbalance the moment of the distalizing force [35]. Moreover, the extrusion of the upper incisors was necessary in the refinement phase to increase the overbite. Savignano et al. [36] found that the rectangular palatal attachment provides bodily movement without palatal inclination of the crown during incisors extrusion, as the line of action of the force passes closer to the center of resistance of the teeth. For this reason, as we needed to avoid a decrease in overjet during the extrusion movement, palatal attachments were chosen.

Another advantage of clear aligners therapy was to be able to create a fully digital workflow with the help of three-dimensional technology in diagnosis, planning, and assessing the impact of therapy on the soft tissues of the face and in evaluating the results in progress [37,38]. The accurate virtual planning of the case ensured that, during the treatment, despite the remoteness, the patient, maintaining the necessary collaboration, achieved the treatment goals and thus only a finishing phase with a few aligners was necessary. After 20 months, initial treatment objectives had been obtained, dental aesthetics significantly improved, and the patient was satisfied with the outcome. 

The limitation of this study is that it is only a case report. Hence, more scientific literature concerning Class III subdivision treatment with the use of clear aligners and an isolated extraction of a bicuspid in the lower arch is needed to improve and validate this technique. More widely, further research in borderline Class III cases treated with clear aligners is necessary to learn more about this issue.

## 4. Conclusions

This case report demonstrated a successful approach to treating Class III subdivision malocclusion in an adult patient using clear aligners. The extraction of a lower bicuspid and Class III elastic on one side only at night led to a satisfactory occlusal and aesthetic correction, with higher predictability and reduced time compared to unilateral molar distalization. An extractive therapy with clear aligners could be considered a valid alternative in Class III non-growing patients who refuse orthognathic surgery and choose a treatment aimed at dentoalveolar compensation.

## Figures and Tables

**Table 1:** Cephalometric measurements before and after treatment.

Measurements	Initial	Final	Norm
SNA (°)	86.7	87.5	82.0 ± 3.5
SNB (°)	84.1	84.9	80.0 ± 3.5
ANB (°)	2.6	2.6	2.0 ± 2.5
SN^ANS-PNS (°)	7.2	6.3	8.0 ± 3.0
SN^GoGn (°)	37.7	37.7	33.0 ± 2.5
ANS/PNS^Go-Gn (°)	33.3	33.3	25.0 ± 6.0
U1^ANS-PNS (°)	113.2	111.8	110.0 ± 6.0
L1^GoGn (°)	85.1	80.4	90.0 ± 6.0
L1 Protrusion (L1-APo) (mm)	1.6	1.3	1.0 ± 2.5
Overjet (mm)	2.6	2.4	2.5 ± 2.5
Overbite (mm)	0.6	1.5	3.0 ± 2.5
U1^L1 (°)	131.1	136.4	135.0 ± 6.0
Co-Go-Me (°)	123.9	124.8	125.0
Lower lip to Ricketts E-line (mm)	−4.8	−5.6	0.0 ± 2.0

## Data Availability

All data generated or analyzed during this study are included in this article.
